# Carpesabrolide A, a novel meroterpenoid with anti-inflammatory activity from *Carpesium abrotanoides*†

**DOI:** 10.1039/d4ra00292j

**Published:** 2024-04-05

**Authors:** Xiao-Fang Zhang, Jiao-Xian Du, Si-Qiong Teng, Hui Liu, Juan He, Tao Feng, Ji-Kai Liu

**Affiliations:** a School of Pharmaceutical Sciences, South-Central Minzu University 182 Minzu Road Wuhan 430074 P.R. China 2015048@mail.scuec.edu.cn tfeng@mail.scuec.edu.cn liujikai@mail.scuec.edu.cn; b National Demonstration Center for Experimental Ethnopharmacology Education, South-Central Minzu University Wuhan 430074 China; c College of Life Science, South-Central Minzu University Wuhan 430074 China

## Abstract

Carpesabrolide A (1), featuring an unprecedented fumaric acid–guaiane sesquiterpenoid hybrid, has been isolated from the folk medicinal plant *Carpesium abrotanoides*. The structure with absolute configuration has been established by spectroscopic methods and single crystal X-ray diffraction analysis. The plausible biosynthetic pathway for 1 is proposed. Compound 1 shows significant anti-inflammatory activity by inhibiting NO production with an IC_50_ value of 2.7 μM.

## Abstract 2

Carpesabrolide A is a fumaric acid–guaiane sesquiterpenoid hybrid isolated from the medicinal plant *Carpesium abrotanoides*. It significantly inhibited NO production with an IC_50_ value of 2.7 μM.

## Graphical abstract

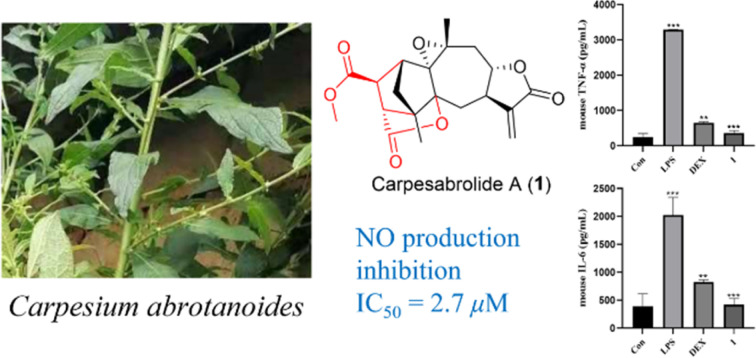

## Introduction

The genus *Carpesium* (Asteraceae) contains about 21 species worldwide.^1,2^ They are mostly perennial herbs and distributed across Asia and Europe, particularly in the mountainous areas of Southwest China.^3^ Many *Carpesium* plants have been used in traditional medicines for the treatments of fevers, colds, bruises, insect and snake bites in China, Korea, Japan and other Asian countries.^4,5^ Previous chemical investigations have demonstrated that *Carpesium* plants are a good source of monoterpenoids,^6,7^ sesquiterpenoids,^8,9^ diterpenoids and phenolic derivatives,^10–12^ with various biological activities such as anti-inflammatory,^13^ anti-tumor,^14^ insecticidal^15^ and bactericidal effects.^16^

*Carpesium abrotanoides* L. is a perennial herb widely distributed in East, central, South and southwest China at altitudes below 2000 m.^17^ Its fruit is used in the insecticidal prescription of traditional Chinese medicine.^1,18^*C. abrotanoides* is also a famous Tujia medicine used for detoxifying toxins, hemostatic, killing worms, tonsillitis, malaria, acute hepatitis and itchy skin rashes for a long time.^19^ According to literature, sesquiterpenes are the main secondary metabolites found in *C. abrotanoides*, including guaiane-type, eudesmane-type, eremophilane-type, *etc.*, with diverse pharmacological properties, such as anti-inflammatory,^20^ antiviral,^21^ antifungal,^22^ antibacterial^23^ and cytotoxic activities.^1,24,25^

**Fig. 1:**
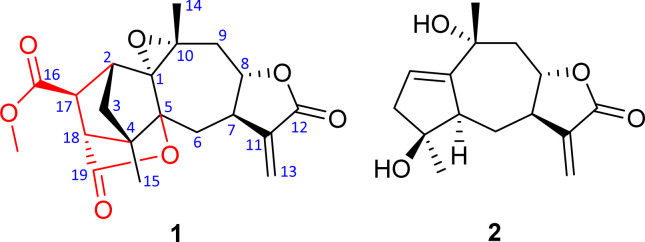
Structures of compounds 1 and 2.

The diversity of structures and biological activities has aroused our great research interest in the chemical composition of *C. abrotanoides*. In this study, a novel succinic acid–guaiane meroterpenoid, namely carpesabrolide A (1), together with its possible precursor compound 2, has been isolated from the whole grass of *C. abrotanoides* (Fig. 1). The anti-inflammatory activity for 1 was tested. Herein, the isolation, structural elucidation, possible biosynthetic pathway, and anti-inflammatory activity of these isolates are reported.

## Results and discussion

The dried and powdered whole grass of *C. abrotanoides* (5 kg) was extracted three times by methanol to give a crude extract (800 g). The crude extract was distributed by EtOAc and H_2_O to give an organic layer (160 g). The latter was separated by column chromatography to give compounds 1 (6 mg) and 2 (12 mg).

Compound 1 was obtained as colorless needles (MeOH). Its molecular formula, C_20_H_22_O_7_, was established on the basis of HRESIMS (measured at *m*/*z* 375.14395 [M + H]^+^; calcd for C_20_H_22_O_7_H^+^, 375.14383), corresponding to ten degrees of unsaturation. The ^1^H NMR data (Table 1) displayed three singlets at *δ*_H_ 1.33, 1.52, and 3.73, which could be readily assigned as two methyls and one methoxy group, respectively. In addition, two olefinic protons at *δ*_H_ 6.16 and 5.68 with a small coupling constant of *J* = 3.2 Hz suggested the presence of a terminal double bond, while one signal at *δ*_H_ 4.19 indicated the presence of oxygenated group(s) in the structure. In the ^13^C NMR spectrum, a total of 20 carbon resonances could be detected (Table 1), which were classified into three CH_3_, four CH_2_, five CH, and eight no-protonated carbons by DEPT and HSQC technologies.

**Table 1:** ^1^H (600 MHz) and ^13^C (150 MHz) NMR data for 1 in methanol-*d*_4_^a^

No.	*δ*_H_	*δ*_C_
1		75.8, C
2	2.6, brs	45.5, CH
3a	1.79, dd (12.1, 1.5)	35.3, CH_2_
3b	1.67, dd (12.1, 1.5)	
4		58.4, C
5		91.3, C
6a	2.37, dd (14.8, 2.0)	33.4, CH_2_
6b	1.84, dd (14.8, 11.5)	
7	2.76, m	45.2, CH
8	4.19, ddd (12.4, 9.3, 3.0)	81.5, CH
9a	2.75, dd (14.0, 3.0)	43.8, CH_2_
9b	2.28, dd (14.0, 12.4)	
10		68.6, C
11		140.9, C
12		171.2, C
13a	6.16, d (3.2)	120.7, CH_2_
13b	5.68, d (3.2)	
14	1.52, s	25.0, CH_3_
15	1.49, s	14.9, CH_3_
16		173.7, C
17	3.13, d (1.9)	50.2, CH
18	2.96, d (1.9)	52.1 CH
19		179.2, C
OMe	3.73, s	53.1, CH_3_

a Data were assigned by HSQC, HMBC, ^1^H–^1^H COSY and ROESY spectra.

**Fig. 2:**
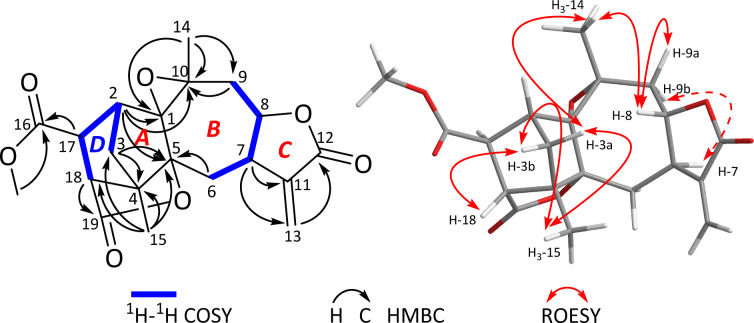
Key 2D NMR correlations of 1.

The ^1^H–^1^H COSY spectrum revealed the presence of two spin systems, including correlations of H_2_-3/H-2/H-17/H-18 (a) and H_2_-6/H-7/H-8/H_2_-9 (b) as showed in Fig. 1. Based on these data, the HMBC experiment established the planar structure of 1. As shown in Fig. 1, the HMBC correlations from H_2_-3 to C-4 and C-5, from H-2 to C-1 and C-5, and from H_3_-15 to C-3, C-4, and C-5 established a five-membered ring *A*, with a methyl substituent at C-4. The HMBC correlations from H_3_-14 to C-1, C-9, and C-10, from H-9 to C-10, and from H_2_-6 to C-1 and C-5 constructed a seven-membered ring *B*, including a methyl group at C-10. Furthermore, the HMBC correlations from H-7 to C-11 and C-13, and from H-13 to C-12 built a γ-lactone *C*, with a conjugated exocyclic double bond. So far, a 5/7/5-fused ring system was established, which resembled a guaiane sesquiterpene backbone, with reference to those in the literature.^26,27^ Further analyses of the NMR and MS data suggested the presence of an epoxy moiety between C-1 and C-10 in ring *B*. Five remaining carbons were assigned to monomethyl fumarate based on the HMBC correlations from *δ*_H_ 3.73 (3H, s, OMe) to *δ*_C_ 173.7 (s, C-16) and from H-17 and H-18 to C-16 and C-19. Importantly, the HMBC correlations from H_3_-15 to C-18 and from H-18 to C-4 established a five-membered ring *D* (Fig. 2). The HMBC correlations from H-17 to C-1, C-3, and C-4, from H-18 to C-5, and H_2_-3 to C-18 suggested that the ring *D* was fused together with the ring *A via* C-2/C-3/C-4. A possible lactone moiety was suggested to be placed between C-5 (*δ*_C_ 91.3, s) and C-19 (*δ*_C_ 179.2, s) according to analyses of the HMBC correlations from H-18 to C-19, C-5, along with MS data analysis. Therefore, the structure of 1 was established as an unprecedented fumaric acid–guaiane meroterpenoid with a complicated polycyclic system.

**Fig. 3:**
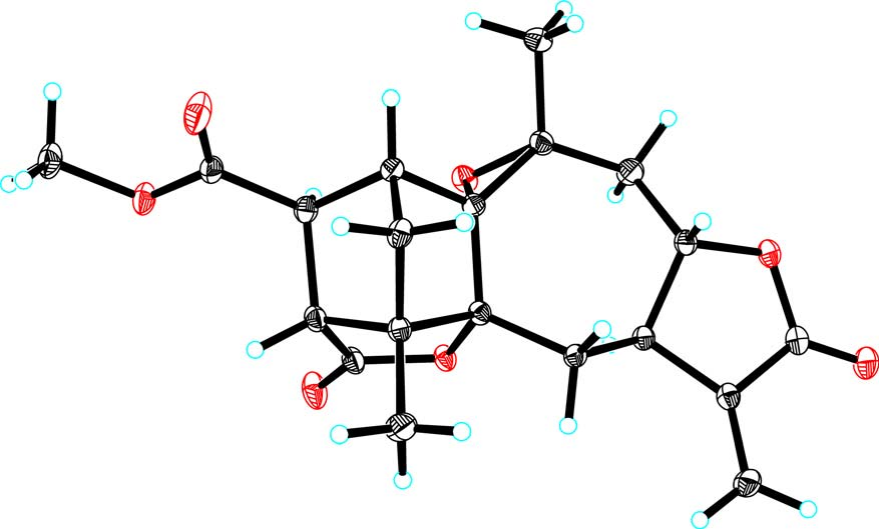
ORTEP drawing for 1 showing absolute configuration.

In the ROESY spectrum (Fig. 2), two key ROE correlations of H_3_-14/H-3a and H_3_-14/H-8 indicated that H_3_-14 and H-8 were on the same face, which were assigned as β-orientation tentatively. Accordingly, the ROE correlations of H-8/H-9a and H-9b H^−1^-7 indicated that H-7 should be α-oriented. In addition, the key ROE correlation between H-18 and H-3b suggested that H-18 to be β-oriented, while the cross peak between H_3_-15 and H-3 allowed the lactone moiety between C-5 and C-19 to be α-oriented. The small coupling constant of *J*_17,18_ = 1.9 Hz suggested the angle between H-7 and H-18 close to 90°, allowing H-17 to be α-oriented. Finally, the single crystal X-ray diffraction (Cu Kα radiation) not only confirmed the planar structure but also clarified the absolute configuration (Fig. 3). Hence, the structure of 1 was identified and trivially named carpesabrolide A.

**Scheme 1:**
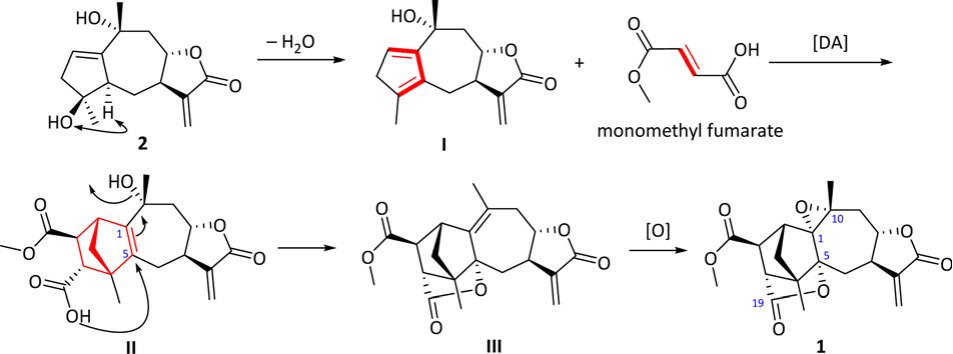
Proposed biosynthetic pathway for 1.

Carpesabrolide A (1) represents hitherto the first example of fumaric acid–sesquiterpene hybrid directly linked by two carbon bonds, which aroused our interest in its plausible biosynthesis pathway. As shown in Scheme 1, compound 2 was isolated in this study, which should be a possible biogenetic precursor of 1. Through dehydration, compound 2 formed a key diene body I, which reacted with monomethyl fumarate through a Diels–Alder cycloaddition to produce II, featuring a novel bicyclo[2.2.1]heptane moiety. Then an epoxide moiety between C-1 and C-10 and a lactone between C-5 and C-19 were established, and compound 1 was finally produced.

**Fig. 4:**
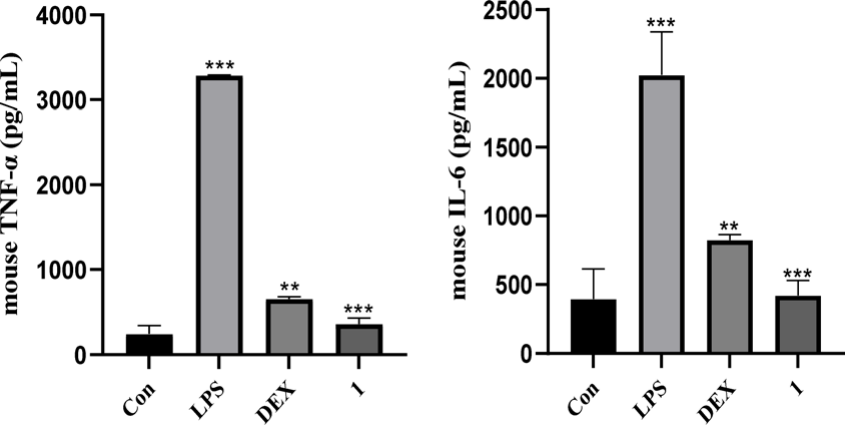
Effects of 1 on LPS-induced pro-inflammatory cytokines production in RAW264.7 cells.

Sesquiterpenoids from *C. abrotanoides* have been widely studied for their pharmacological activities, and many of them have been demonstrated promising anti-inflammatory activity.^20^ Therefore, compound 1 was evaluated for its anti-inflammatory activity by inhibiting NO release in LPS-activated RAW264.7 cells. As a result, compound 1 showed significant inhibitory activity against NO production with an IC_50_ value of 2.7 μM, better than that of dexamethasone (positive control, IC_50_ = 9.0 μM). Furthermore, the effects of 1 on pro-inflammatory mediators *TNF-α* and *IL-6* were investigated in LPS-stimulated RAW264.7 cells using enzyme linked immunosorbent assay (ELISA) as we reported previously.^28^ As depicted in Fig. 4, the results suggested that compound 1 visibly suppressed the secretion of *TNF-α* and *IL-6* compared to the LPS-only treatment at the concentration of 5 μM, better than that of dexamethasone (10 μM) as well.

## Conclusions

In conclusion, carpesabrolide A (1), an unprecedented fumaric acid–guaiane sesquiterpenoid hybrid, was isolated from the traditional Chinese medicine *C. abrotanoides*. The novel carbon skeleton and significant anti-inflammatory activity make it a good scaffold for further chemistry study and biological investigation. Although the chemical composition and biological activity of *C. abrotanoides* have been largely studied previously, the discovery in study suggested that there are still rich resources for this medicinal plant and it is worth exploring further.

## Experimental section

### General experimental procedures

Melting points were measured on a WRX-4 apparatus. Optical rotations were measured with a Horiba SEPA-300 polarimeter. IR spectra were obtained with a Tenor 27 spectrophotometer using KBr pellets. 1D and 2D spectra were run on a Bruker Avance III 600 MHz spectrometer with TMS as an internal standard. Chemical shifts (*δ*) were expressed in ppm with reference to the solvent signals. Mass spectra were recorded on an Agilent 6200 Q-TOF mass spectrometry system. Column chromatography (CC) was performed on silica gel (200–300 mesh), RP-18 gel (20–45 μm), and Sephadex LH-20. Medium pressure liquid chromatography (MPLC) was performed on a Biotage One equipment packed with RP-18 gel columns. Preparative high performance liquid chromatography (prep-HPLC) was performed on an Agilent 1260 liquid chromatography system equipped with Zorbax SB-C_18_ columns (5 μM, 9.4 × 250 mm) and a DAD detector. Fractions were monitored by TLC (GF 254), and spots were visualized by heating silica gel plates sprayed with 10% H_2_SO_4_ in EtOH.

### Plant material

The whole plant of *Carpesium abrotanoides* L. was collected from Yunnan, China, in August 2022, and identified by Mr Jun Zhang. A voucher specimen (HFG-P-CA20220812.2) was deposited at School of Pharmaceutical Sciences, South-Central Minzu University.

### Extraction and isolation

The dried and powdered whole grass of *C. abrotanoides* (5 kg) was extracted three times by methanol to obtain a crude extract (800 g). The crude extract was further extracted with EtOAc to give an organic layer (160 g). The latter was separated by CC over silica gel eluted with CH_2_Cl_2_/MeOH (from 1 : 0 to 0 : 1) to obtain eight fractions (A–H). Fraction C (8.1 g) was further separated by CC over silica gel eluted with CH_2_Cl_2_/MeOH (12 : 1) to give seven subfraction C1–C7. Fraction C6 (620 mg) was subjected MPLC (MeOH/H_2_O, from 2 : 8 to 8 : 2) to give eight subfractions C6_1_–C6_8_. Fraction C6_7_ (85 mg) was purified by HPLC (MeCN/H_2_O, from 3 : 7 to 4 : 6 in 30 min) to give compound 1 (4 mg, *t*_R_ = 24 min). Fraction D (2.2 g) was subjected to MPLC to give seven subfractions D1–D7. Fraction D4 (490 mg) was purified by HPLC (MeCN/H_2_O, from 3 : 7 to 4 : 6 in 35 min) to give compound 2 (12 mg, *t*_R_ = 26 min).

#### Carpesabrolide A (1)

White crystals; mp 285–287 °C, [*α*]^23^_D_ +18 (*c* 0.5, MeOH); UV (MeOH): *λ*_max_ (log *ε*): 210 (2.87) nm; ^1^H NMR (600 MHz, methanol-*d*_4_) and ^13^C NMR (150 MHz, methanol-*d*_4_) data in Table 1, respectively; HRESIMS (Positive) *m*/*z* 375.14383 [M + H]^+^ (calcd for C_20_H_23_O_7_, 375.14395).

#### Crystal data for carpesabrolide A (1)

C_20_H_22_O_7_, *M* = 374.37, *a* = 9.8989(3) Å, *b* = 11.3680(3) Å, *c* = 15.8912(5) Å, *α* = 90°, *β* = 90°, *γ* = 90°, *V* = 1788.25(9) Å^3^, *T* = 150(2) K, space group *P*2_1_2_1_2_1_, *Z* = 4, *μ*(Cu Kα) = 0.881 mm^−1^, 16 042 reflections measured, 3367 independent reflections (*R*_int_ = 0.0552). The final *R*_1_ values were 0.0295 (*I* > 2*σ*(*I*)). The final w*R*(*F*^2^) values were 0.0723 (*I* > 2*σ*(*I*)). The final *R*_1_ values were 0.0310 (all data). The final w*R*(*F*^2^) values were 0.0731 (all data). The goodness of fit on *F*^2^ was 1.057. Flack parameter = 0.08(7). The crystal data (cif file) was deposited at the Cambridge Crystal Data Center (CCDC) (http://www.ccdc.cam.ac.uk) under access number 2311963.†

### *In vitro* anti-inflammatory activity

#### Determination of NO production

NO production was measured indirectly using supernatant. RAW264.7 cells were seeded in 96-well plates and cultured for 24 h, then the medium was changed and treated with sample solutions at concentrations of 1, 5, 10, 20, 30, 40 μM for 1 h. The IC_50_ of each tested compound was determined by collecting the supernatant after 12 h of incubation with the addition of LPS, which was used to measure NO production.

#### Determination of pro-inflammatory cytokine (TNF-α and IL-6)

RAW264.7 cells were inoculated into 96-well plates at a density of 1 × 10^5^ cells per well and cultured overnight. Then, the supernatant was collected after treating the cells with LPS (1 μg mL^−1^) or LPS (1 μg mL^−1^) + DEX (10 μM) or LPS (1 μg mL^−1^) + 1 (5 μM) for 24 h. The concentrations of *TNF-α* and *IL-6* in the supernatant were measured using ELISA kits according to the manufacturer's instructions (Neobioscience, Shenzhen, China). Briefly, the plates were removed and equilibrated to room temperature. The samples were diluted to the desired concentration, then added to the plates and incubated at 37 °C for 90 min. The plates were then washed three times, then incubated at 37 °C with the biological antibody, enzyme conjugate, and colorant for 60, 30, and 15 min, respectively. Termination solution was added, and the absorbance was measured at 450 nm within 3 min. Finally, the concentrations of *TNF-α* and *IL-6* were calculated from the standard curves.

## Author contributions

Xiao-Fang Zhang contributed to the isolation and structure elucidation. Jiao-Xian Du and Si-Qiong Teng contributed to the biological activity test. Hui Liu and Juan He contributed to structure determination and X-ray diffraction. Tao Feng guided the experiments and the manuscript preparation. Ji-Kai Liu designed the experiments.

## Conflicts of interest

There are no conflicts to declare.

## Supplementary Material

RA-014-D4RA00292J-s001

RA-014-D4RA00292J-s002
