# Supplementary material for: Carpesabrolide A, a novel meroterpenoid with anti-inflammatory activity from Carpesium abrotanoides
Source: RSC Adv. 2024 Apr 5;14(16):11002–6. doi: 10.1039/d4ra00292j (PMC10995668; doi:10.1039/d4ra00292j)
Supplement: RA-014-D4RA00292J-s001 [file RA-014-D4RA00292J-s001.pdf]

Electronic Supplementary Material (ESI) for RSC Advances.
This journal is © The Royal Society of Chemistry 2024

Supporting information for

Carpesabrolide A, a novel meroterpenoid with anti-inflammatory activity from *Carpesium abrotanoides*

Xiao-Fang Zhang, Jiao-Xian Du, Si-Qiong Teng, Hui Liu, Juan He,* Tao Feng* and Ji-Kai Liu*

College of Life Science, and School of Pharmaceutical Sciences, South-Central Minzu University, Wuhan 430074, China

*Corresponding authors: liujikai@mail.scuec.edu.cn (J. K. Liu), tfeng@mail.scuec.edu.cn (T. Feng), 2015048@mail.scuec.edu.cn

Content

S1. NMR spectra and MS for carpesabrolide A (1) S2

S2. NMR spectra for 4*β*,10*α*-dihydroxy-5*α*(H)-1,11(13)-guaidien-8*α*,12-olide (2) S9

S3. X-ray crystal data for carpesabrolide A (1) S11

S1. NMR spectra and MS for carpesabrolide A (1)

^{1}H NMR of **1** in methanol-d_4

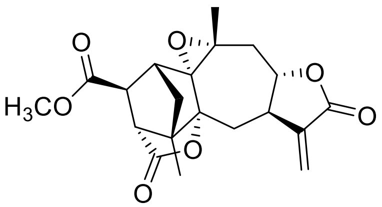

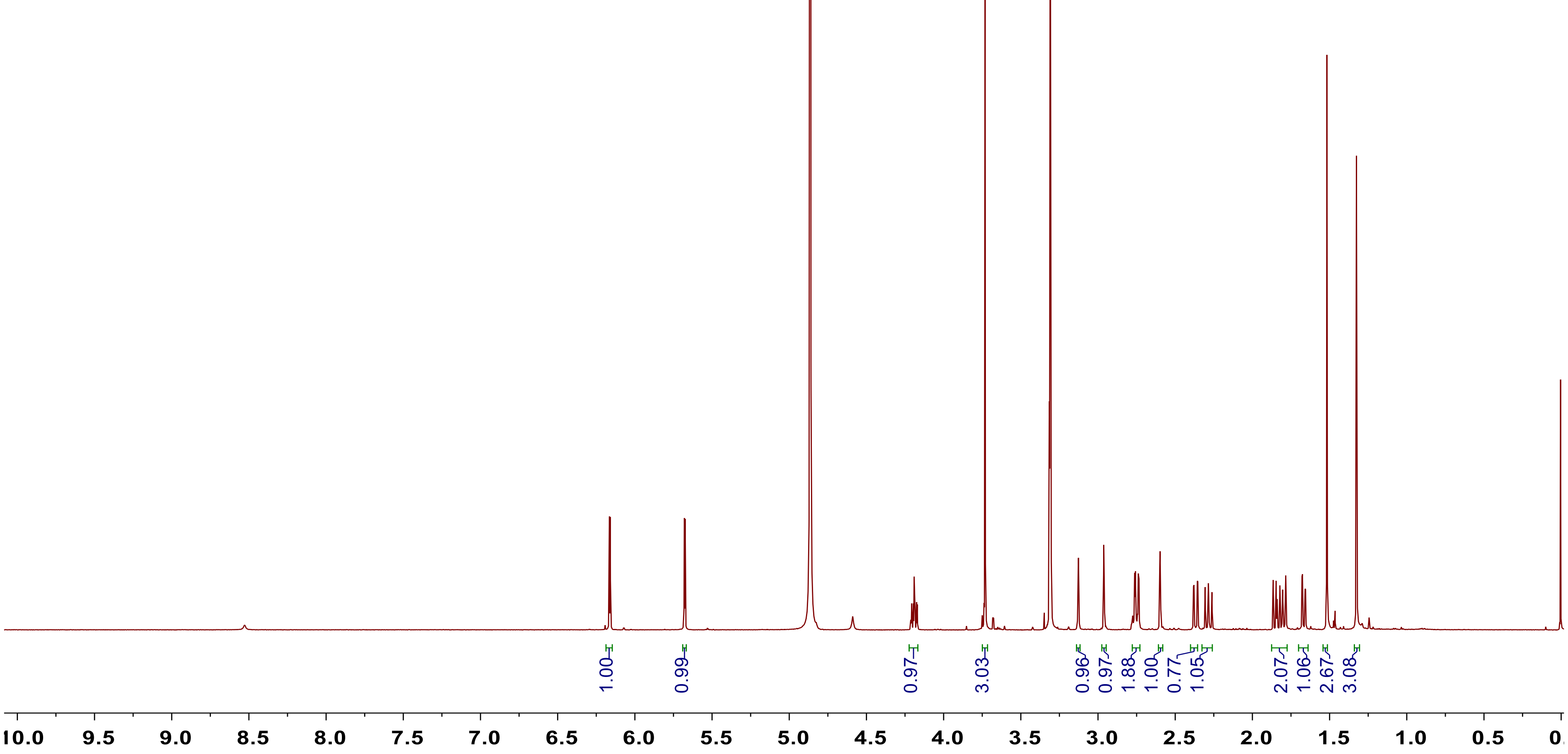

^{13}C NMR and DEPT of **1** in methanol-d_4

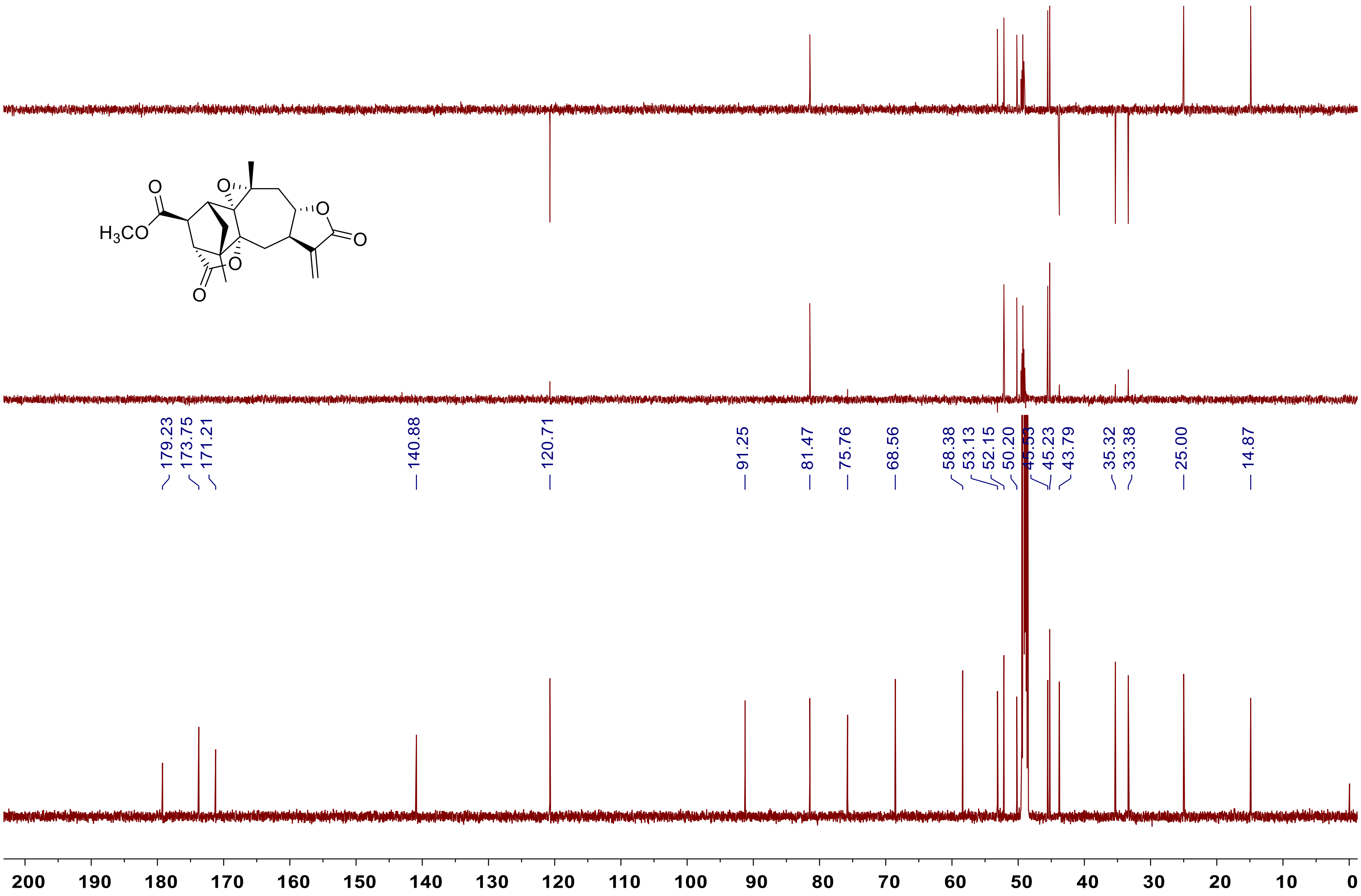

HSQC of **1** in methanol-d_4

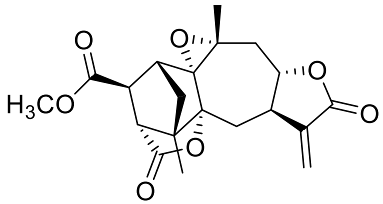

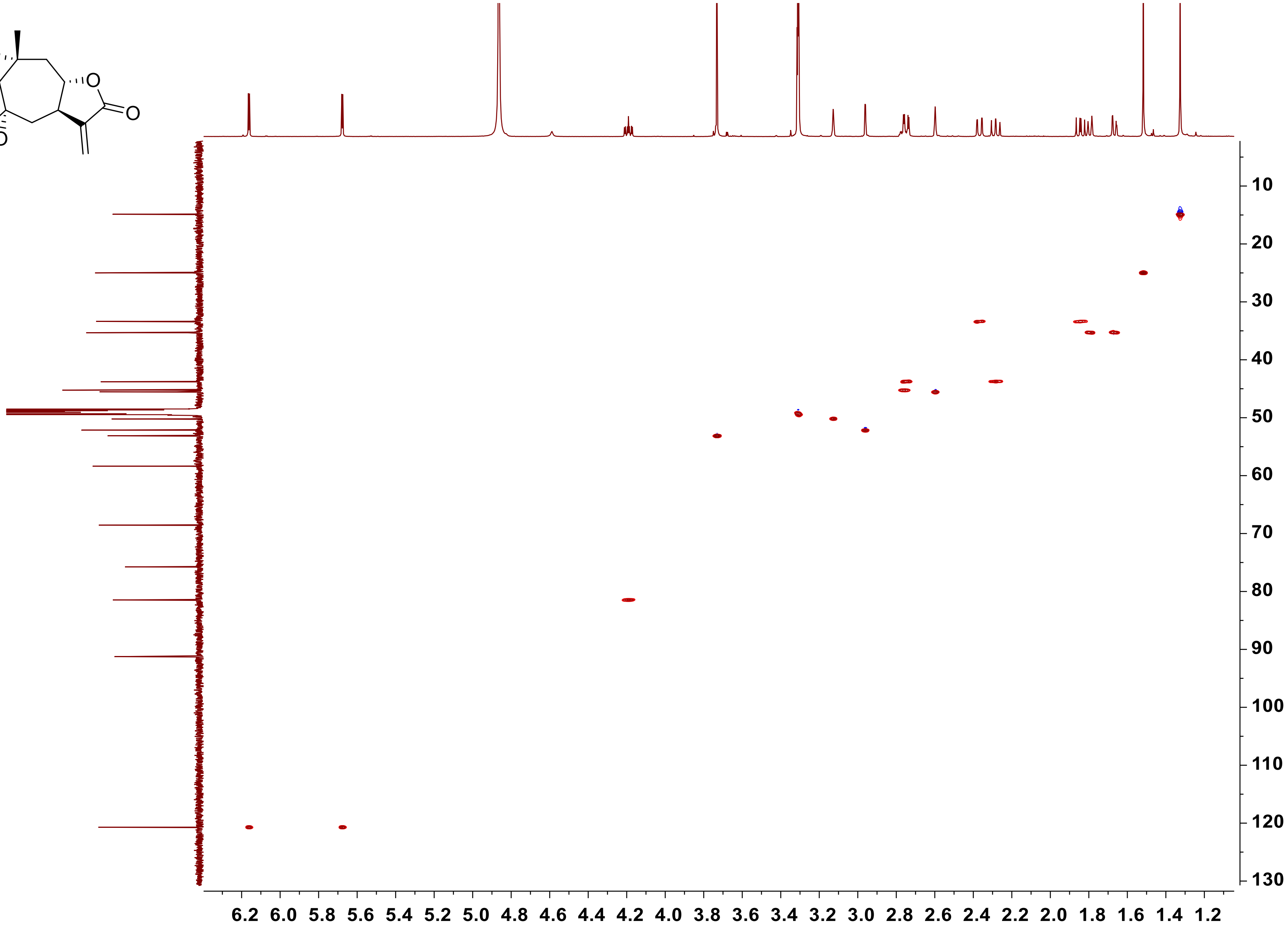

HMBC of **1** in methanol-d_4

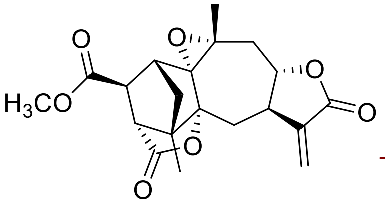

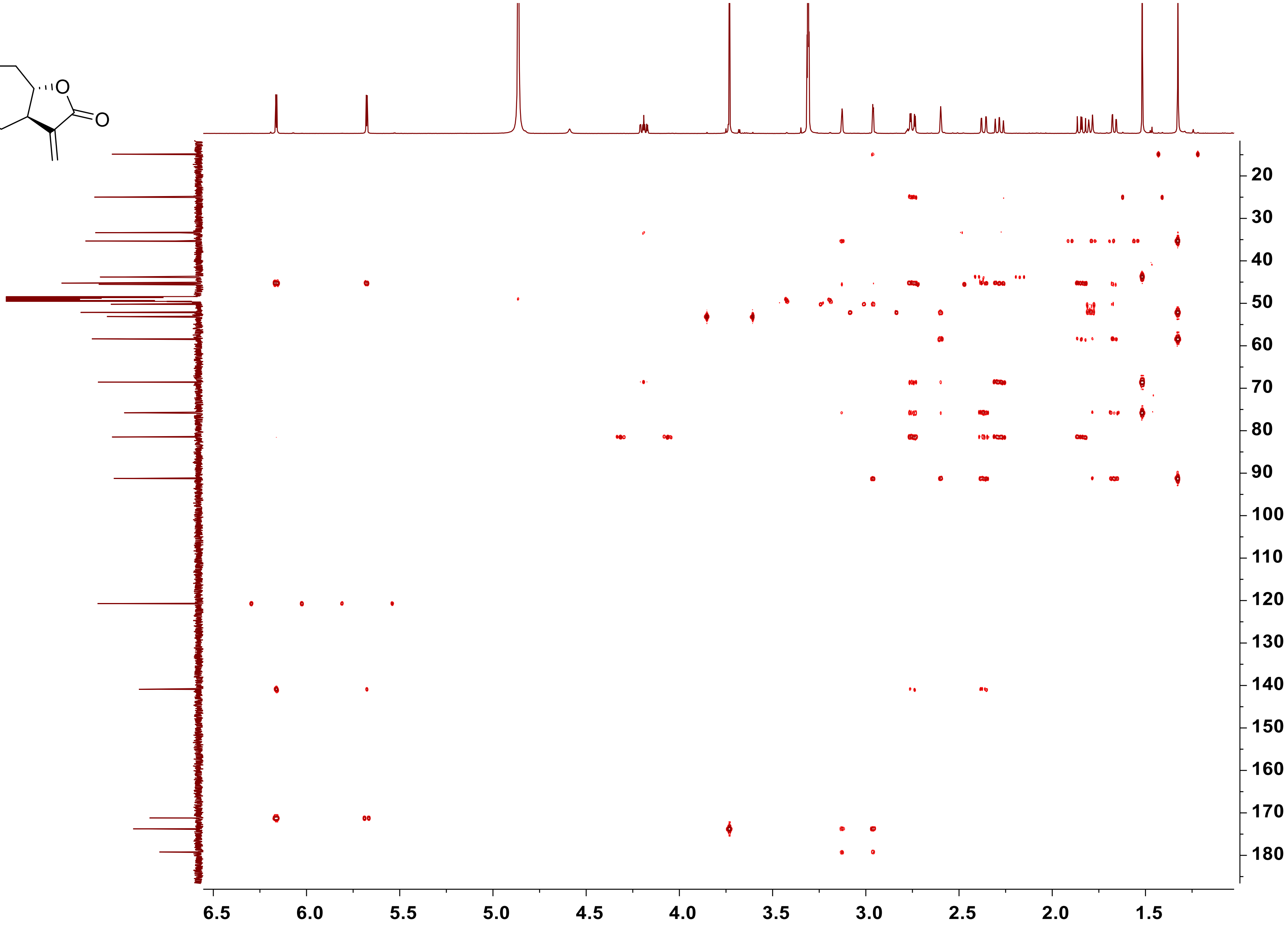

^{1}H-^{1}H COSY of **1** in methanol-d_4

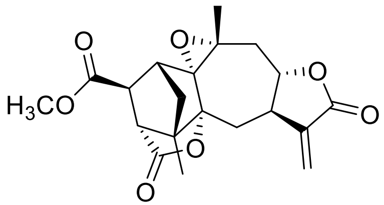

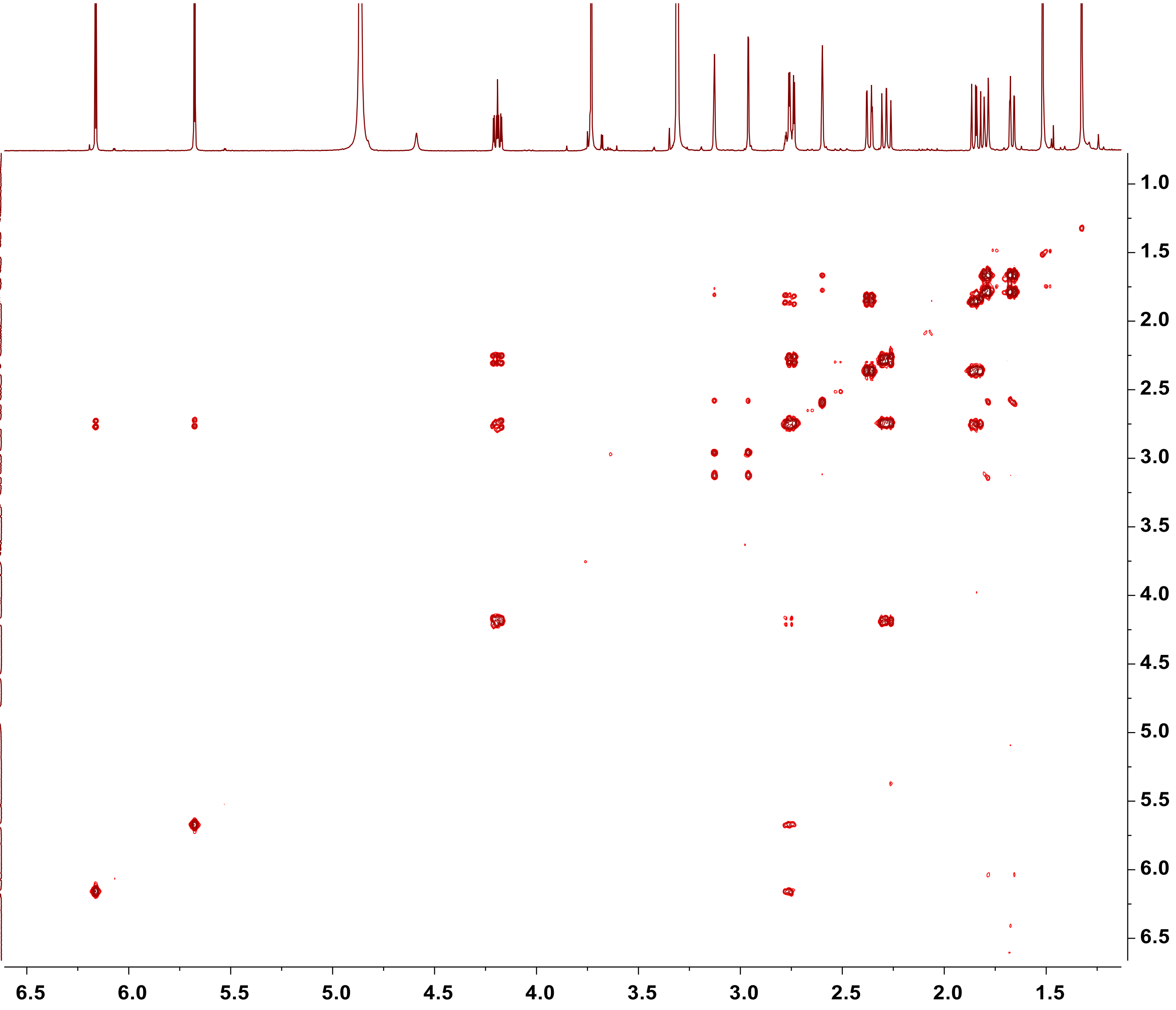

ROSEY of **1** in methanol-d_4

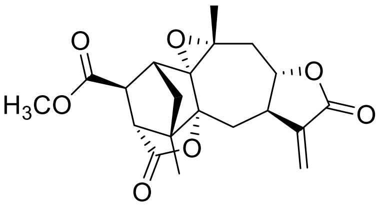

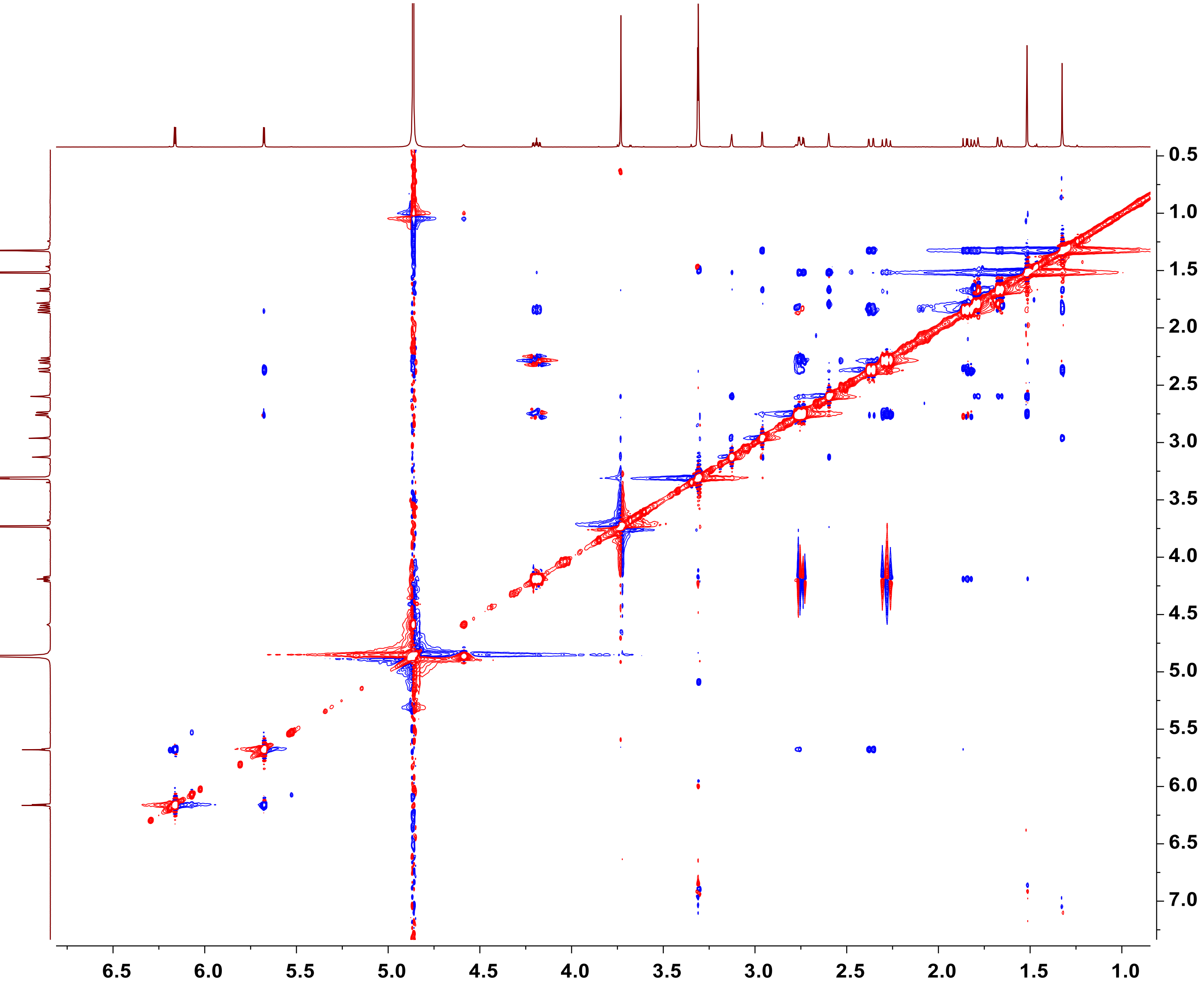

HR-ESIMS of **1**

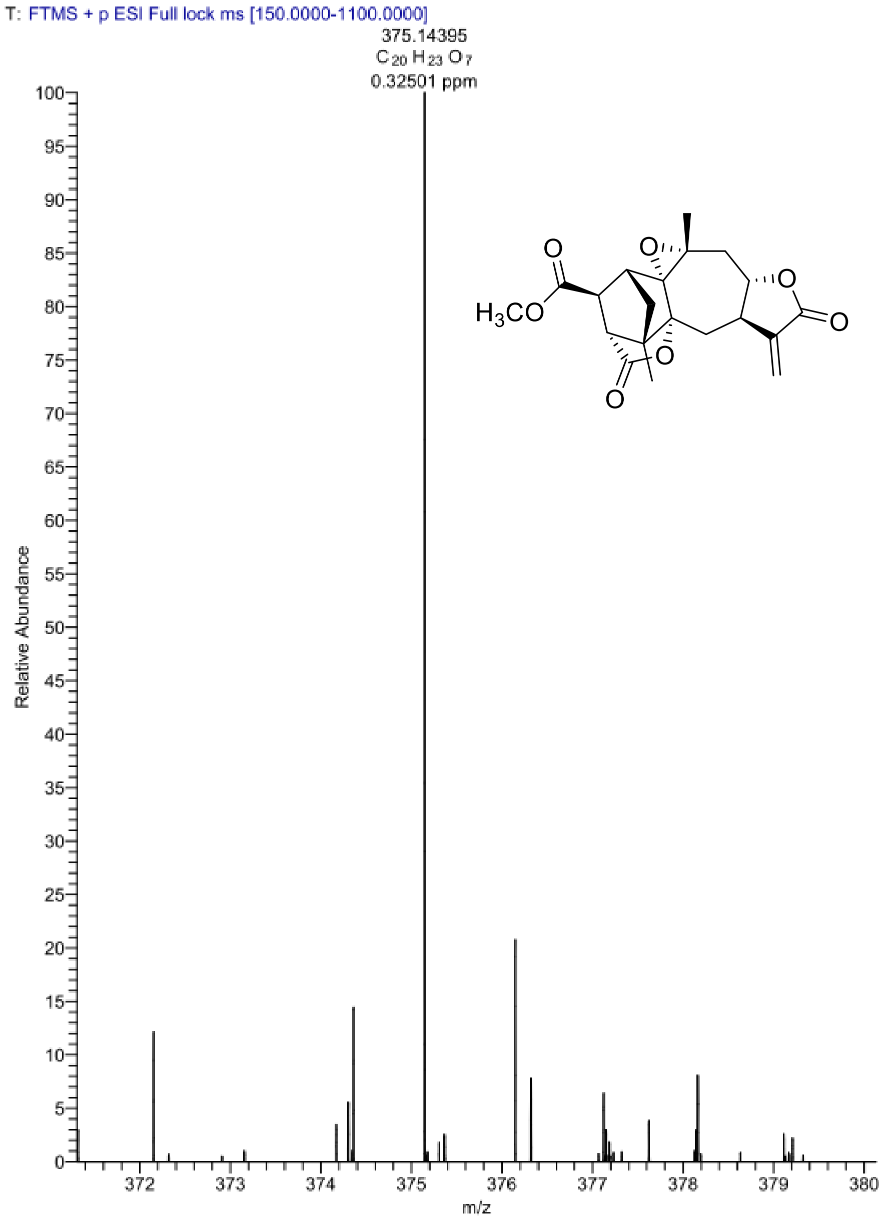

T: FTMS + p ESI Full lock ms [150.0000-1100.0000]
375.14395
C 20 H 23 O 7
0.32501 ppm
Relative Abundance
m/z
H3CO

S1. NMR spectra for 4*β*,10*α*-dihydroxy-5*α*(H)-1,11(13)-guaidien-8*α*,12-olide (2)

^{1}H NMR (500 MHz) of **2** in methanol-d_4

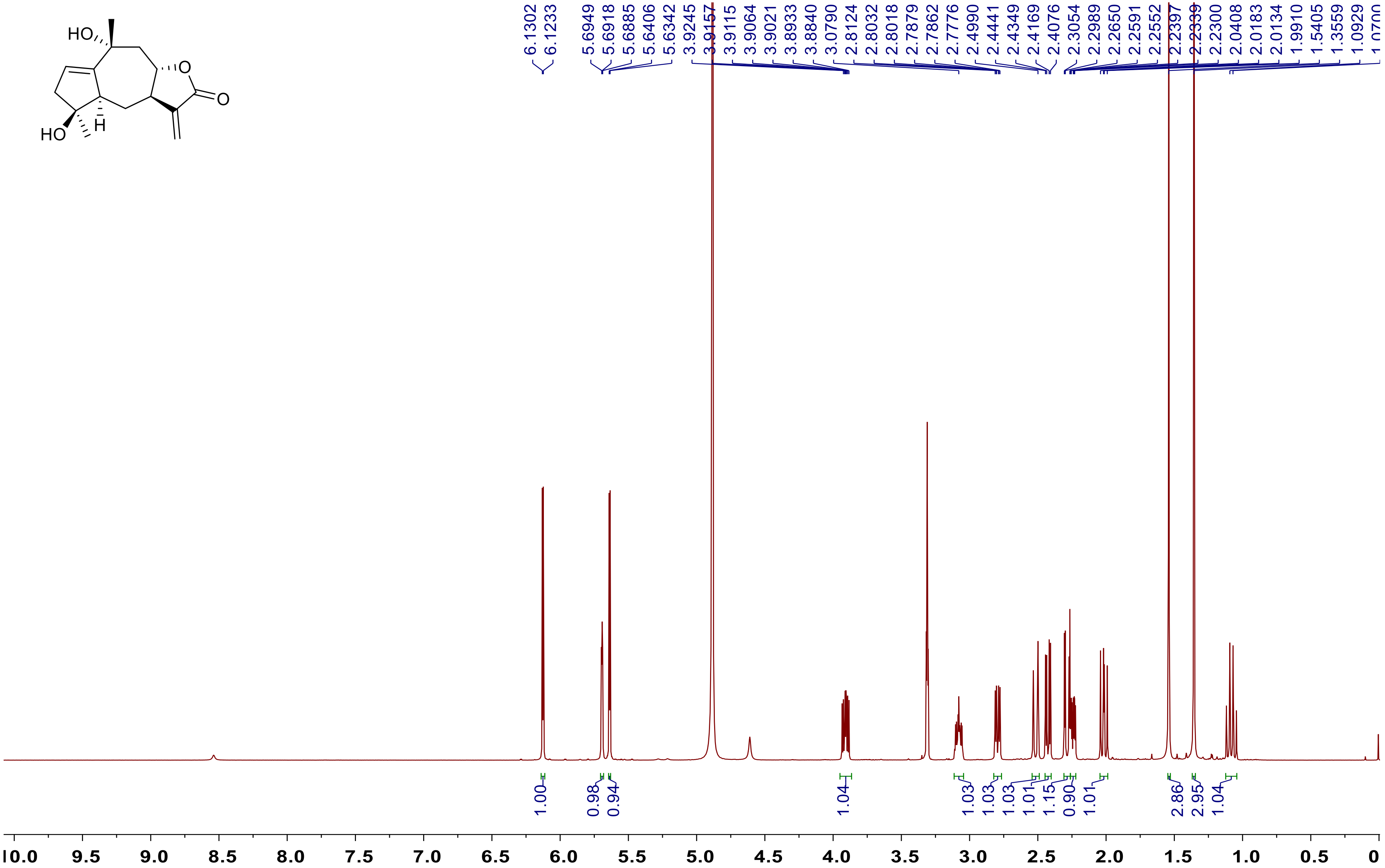

^{13}C NMR and DEPT (125 MHz) of **2** in methanol-d_4

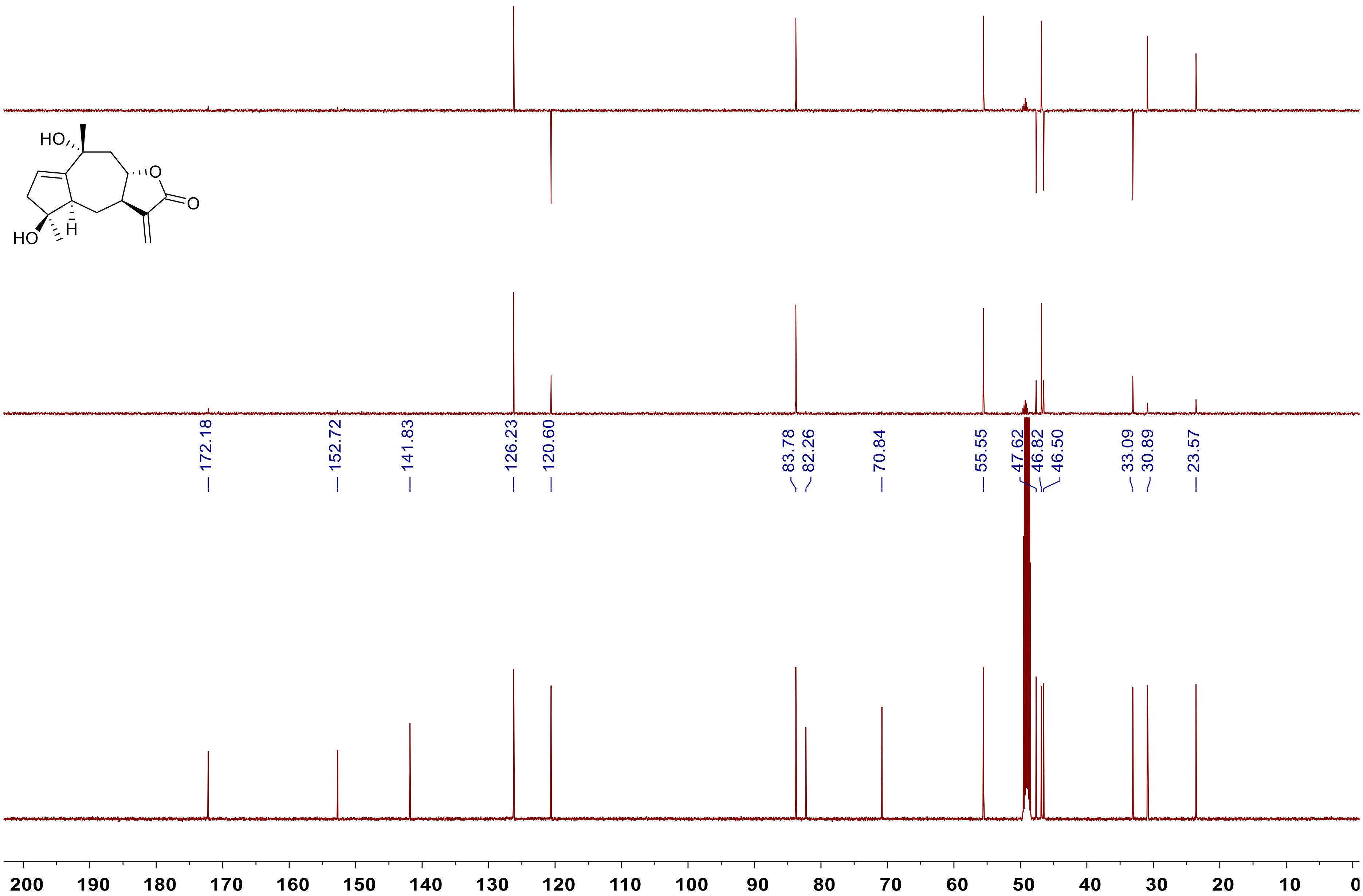

S2. X-ray crystal data for carpesabrolide A (1)

$C_{20}H_{22}O_7$, M = 374.37, a = 9.8989(3) Å, b = 11.3680(3) Å, c = 15.8912(5) Å, α = 90°, β = 90°, γ = 90°, V = 1788.25(9) Å^3, T = 150.(2) K, space group P212121, Z = 4, μ(Cu Kα) = 0.881 mm^{-1}, 16042 reflections measured, 3367 independent reflections (R_{int} = 0.0552). The final R_1 values were 0.0295 ($I > 2\sigma(I)$). The final $wR(F^2)$ values were 0.0723 ($I > 2\sigma(I)$). The final R_1 values were 0.0310 (all data). The final $wR(F^2)$ values were 0.0731 (all data). The goodness of fit on F^2 was 1.057. Flack parameter = 0.08(7).

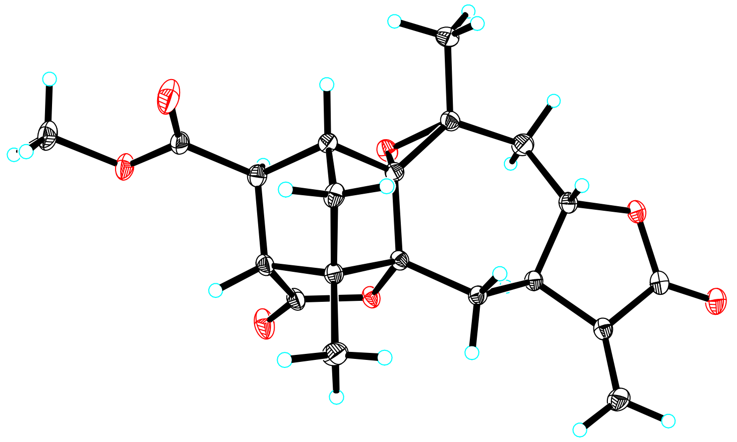

View of a molecule of **1** (displacement ellipsoids are drawn at the 30% probability level)

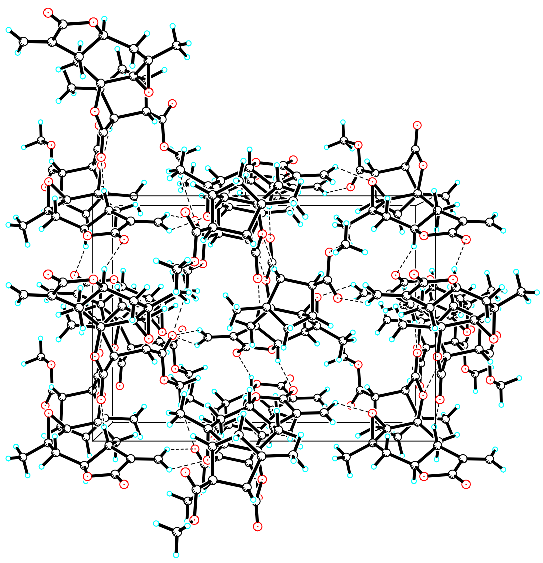

View of the pack drawing of **1** (hydrogen-bonds are shown as dashed lines).

Crystal data and structure refinement for **1**.

Identification code	global	
Empirical formula	$C_{20} H_{22} O_7$	
Formula weight	374.37	
Temperature	150(2) K	
Wavelength	1.54178 Å	
Crystal system	Orthorhombic	
Space group	$P2_12_12_1$	
Unit cell dimensions	a = 9.8989(3) Å	α= 90°.
	b = 11.3680(3) Å	β= 90°.
	c = 15.8912(5) Å	γ = 90°.
Volume	1788.25(9) $Å^3$	
Z	4	
Density (calculated)	1.391 Mg/m^3	
Absorption coefficient	0.881 mm^{-1}	
F(000)	792	
Crystal size	0.360 x 0.200 x 0.140 mm^3	
Theta range for data collection	4.78 to 70.04°.	
Index ranges	-12<=h<=12, -13<=k<=12, -19<=l<=19	
Reflections collected	16042	
Independent reflections	3367 [R(int) = 0.0552]	
Completeness to theta = 70.04°	99.5 %	
Absorption correction	Semi-empirical from equivalents	
Max. and min. transmission	0.89 and 0.72	
Refinement method	Full-matrix least-squares on F^2	
Data / restraints / parameters	3367 / 0 / 247	
Goodness-of-fit on F^2	1.057	
Final R indices [I>2sigma(I)]	R1 = 0.0295, wR2 = 0.0723	
R indices (all data)	R1 = 0.0310, wR2 = 0.0731	
Absolute structure parameter	0.08(7)	
Largest diff. peak and hole	0.183 and -0.184 e.$Å^{-3}$	
